# Plasma exchange and glucocorticoid dosing in the treatment of anti-neutrophil cytoplasm antibody associated vasculitis (PEXIVAS): protocol for a randomized controlled trial

**DOI:** 10.1186/1745-6215-14-73

**Published:** 2013-03-14

**Authors:** Michael Walsh, Peter A Merkel, Chen Au Peh, Wladimir Szpirt, Loïc Guillevin, Charles D Pusey, Janak deZoysa, Natalie Ives, William F Clark, Karen Quillen, Jeffrey L Winters, Keith Wheatley, David Jayne

**Affiliations:** 1 Departments of Medicine and Clinical Epidemiology & Biostatistics, Marian Wing, Division of Nephrology, McMaster University, St. Joseph's Hospital, 50 Charlton Ave East, Hamilton, ON L8S 4A6, Canada; 2 Division of Rheumatology, University of Pennsylvania School of Medicine, Philadelphia, PA, USA; 3 Department of Renal Medicine, Royal Adelaide Hospital, University of Adelaide, Adelaide, Australia; 4 Department of Nephrology, Copenhagen University Hospital, Rigshospitalet, Copenhagen, Denmark; 5 Höpital Cochin, Assistance Publique, Hopitaux de Paris, Universite Paris Descartes, Paris, France; 6 Department of Medicine, Imperial College London, Hammersmith Hospital, London, UK; 7 Department of Renal Medicine, North Shore Hospital, Waitemata District Health Board, Auckland, New Zealand; 8 Birmingham Clinical Trials Unit, University of Birmingham, Birmingham, UK; 9 London Health Sciences Centre, Western University, London, ON, Canada; 10 Department of Medicine, Boston Medical Center, Boston University School of Medicine, Boston, MA, USA; 11 Department of Laboratory Medicine and Pathology, Mayo Clinic College of Medicine, Mayo Clinic, Rochester, MN, USA; 12 Cancer Research UK Clinical Trials Unit, School of Cancer Sciences, University of Birmingham, Birmingham, UK; 13 Lupus and Vasculitis Clinic, Addenbrooke's Hospital, Cambridge, UK

**Keywords:** Factorial trial, Randomized controlled trial, Protocol, Vasculitis, Granulomatosis with polyangiitis, Microscopic polyangiitis, ANCA

## Abstract

**Background:**

Granulomatosis with polyangiitis (GPA, Wegener’s) and microscopic polyangiitis (MPA) are small vessel vasculitides collectively referred to as anti-neutrophil cytoplasm antibody-associated vasculitis (AAV). AAV is associated with high rates of morbidity and mortality due to uncontrolled disease and treatment toxicity. Small randomized trials suggest adjunctive plasma exchange may improve disease control, while observational evidence suggests that current oral glucocorticoid doses are associated with severe infections in patients with AAV. A randomized study of both plasma exchange and glucocorticoids is required to evaluate plasma exchange and oral glucocorticoid dosing in patients with AAV.

**Methods/design:**

PEXIVAS is a two-by-two factorial randomized trial evaluating adjunctive plasma exchange and two oral glucocorticoid regimens in severe AAV. Five hundred patients are being randomized at centers across Europe, North America, Asia, and Australasia to receive plasma exchange or no plasma exchange, and to receive standard or reduced oral glucocorticoid dosing. All patients receive immunosuppression with either cyclophosphamide or rituximab. The primary outcome is the time to the composite of all-cause mortality and end-stage renal disease.

PEXIVAS is funded by the National Institute of Health Research (UK), the Food and Drug Administration (USA), the National Institutes of Health (USA), the Canadian Institute of Health Research (Canada), the National Health and Medical Research Council (Australia), and Assistance Publique (France). Additional in-kind supplies for plasma exchange are provided by industry partners (TerumoBCT, Gambro Australia, and Fresenius Australia).

**Discussion:**

This is the largest trial in AAV undertaken to date. PEXIVAS will inform the future standard of care for patients with severe AAV. The cooperation between investigators, funding agencies, and industry provides a model for conducting studies in rare diseases.

**Trial registration:**

Current Controlled Trials: (ISRCTN07757494) and clinicaltrials.gov: (NCT00987389)

## Background

Granulomatosis with polyangiitis (GPA, Wegener’s) and microscopic polyangiitis (MPA) are syndromes of primary systemic vasculitis associated with anti-neutrophil cytoplasm antibodies (ANCA). Together, these syndromes are grouped as ANCA-associated systemic vasculitis (AAV). The prevalence of AAV is 14 to 30 patients per 100,000 [1]. The most common severe AAV manifestations are glomerulonephritis, leading to renal failure and alveolar capillaritis causing lung hemorrhage.

The current standards of care for initial treatment are either combination cyclophosphamide (CYC) and glucocorticoid (GC) therapy or rituximab and GC therapy. Although these treatments have significantly improved survival compared to untreated AAV, overall survival is still poor and many patients suffer from chronic morbidity including end-stage renal disease (ESRD) [2,3].

Poor survival and ESRD in AAV are attributed both to ineffective therapies and treatment toxicity [2]. At least 20% of patients do not achieve disease control or are intolerant of initial treatment, and an additional 50% will relapse by 5 years [4,5]. Inadequate disease control results in increased immunosuppressive exposure and risk of treatment-related toxicity, progressive organ scarring, and ultimately death [6]. CYC and GC are also associated with high rates of early mortality due to infection [7]. Between 25% and 50% of patients with severe AAV experience a severe infection within the first 12 months of treatment and the most frequently cited causes of death are infection or uncontrolled vasculitis [8,9]. Strategies that improve disease control and reduce toxicity early in treatment will likely have the largest impact on survival, rates of ESRD, and subsequent disease course. PEXIVAS will evaluate two therapies, one to improve early disease control and one to limit early toxicity.

Plasma exchange (PLEX) was introduced into the treatment of pauci-immune glomerulonephritis following its efficacy in the related anti-glomerular basement membrane (GBM) disease, a disease with renal and pulmonary vasculitis induced by pathogenic anti-GBM antibodies [10]. The subsequent discovery of ANCA, their close association with GPA and MPA, and animal models supporting the concept of ANCA as pathogenic antibodies, has provided a rationale for PLEX in AAV. Additional potential beneficial effects of PLEX in AAV include removal of other mediators of inflammation and coagulation, and effects on immunoregulation. Furthermore, small trials comparing PLEX to standard treatments suggest benefits for patients with kidney involvement at presentation [11-13]. Observational work also suggests those with highly active AAV are more likely to benefit from PLEX than those with more chronic presentations [14,15].

Lung hemorrhage is among the most common vasculitis-related causes of early death in AAV and PLEX is widely used for this presentation. This practice comes from cohort data in AAV and extrapolated experience with anti-GBM disease [16-18]. However, PLEX has the potential to exacerbate hemorrhage through removal of clotting factors and increases the risk of infection through antibody removal. Furthermore, the observational data most commonly cited focus on non-contemporaneous patients often with severe manifestations of lung hemorrhage [16]. Contemporary patients are usually diagnosed earlier in their disease course due to the availability of ANCA testing, heightened awareness of the disease, and more routine use of CT scanning, bronchoscopy, and broncho-alveolar lavage.

PLEX is invasive, expensive, labor intensive, and associated with adverse events. There is insufficient high-quality evidence supporting the use of PLEX in AAV, although there are promising data suggesting PLEX may improve survival and prevent ESRD in AAV [8,12]; if this is true, PLEX may be a valuable and cost-effective treatment. Due to the uncertainties surrounding the use of PLEX in AAV, there is a need for a randomized controlled trial examining the effect of PLEX on the important clinical outcomes of ESRD and all-cause mortality.

Oral GC are used ubiquitously in the early management of AAV. There is a complex relationship between GC dose and its effects on the immune system as an immunosuppressive *versus* an anti-inflammatory agent [19]. When combined with cytotoxic medications, high-dose GC may significantly increase treatment-related toxicity while adding little to therapeutic efficacy. Laboratory data suggest lower GC doses may mitigate their toxicity while maintaining anti-inflammatory effects [20]. However, there is often a reluctance to reduce GC doses due to their perceived efficacy at higher doses and the association of the disease with poor outcomes when it is not adequately controlled.

Infections in AAV are most common in the first 6 months of treatment when GC doses are highest [8]. Although this relationship is confounded by disease activity and co-treatment with CYC, it is important to note that infection rates fall in parallel with decreasing GC dose despite the maintenance of constant immunosuppression over time. Replacement of CYC by rituximab has not reduced early infection rates in severe AAV, a finding that supports a major role of GC in infective risk [9,21]. The concept that higher doses of GC increase toxicity without improving treatment efficacy in immunologic diseases is supported from evidence in rheumatoid arthritis and lupus nephritis where a dose-dependent increase in infections is observed with increasing GC dose [22-24]. Furthermore, high cumulative doses of GC are associated with osteoporosis, infections, cardiovascular disease, and gastrointestinal bleeding [22]. In renal transplantation, concern over GC toxicity prompted early GC withdrawal and even GC avoidance regimens. Meta-analyses of these trials have shown that protocols utilizing more rapid GC withdrawal have not lead to increases in ESRD or death [25,26]. Despite the association between higher GC doses and adverse events and despite their widespread use, there is a paucity of literature to guide the optimal use of GC in AAV.

AAV is a rare disease that has ‘orphan’ status in both the US and EU. There are extensive challenges to conducting large, randomized, controlled trials in rare diseases including the need for a large number of study sites to achieve recruitment goals, usually many years for the recruitment period, higher costs per patient compared to studies of common diseases, relative disinterest on the part of the biomedical industry to address clinical problems with small market potential, few drugs with existing indications for a rare disease, and skepticism by funding agencies of the feasibility of such studies [27]. Conducting adequately powered trials in rare diseases requires establishment of research alliances and ‘buy-in’ by a large number of investigators. Collaborative networks of vasculitis investigators have developed in Europe and North America and have established a track record in completing clinical trials and methodologies for their conduct.

There is a need for therapies for AAV with both reduced toxicity and improved disease control. Defining the role of therapies that are already in use but unproven is a priority for research in AAV. PEXIVAS is a randomized clinical trial testing two interventions in a two-by-two factorial design (standard care and PLEX compared to standard care alone and a standard dose GC compared to a reduced dose GC regimen) to address these issues (ISRCTN # ISRCTN07757494; NCT #NCT00987389).

## Methods/design

**Figure 1:**
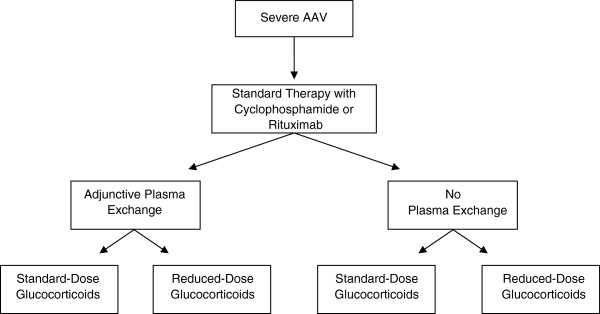
General schema of treatment allocation.

PEXIVAS is an international, open-label, two-by-two factorial design, randomized controlled trial examining: (1) the effect of adjunctive PLEX on the composite end-point of death/ESRD; and (2) the effect of reduced compared to standard glucocorticoids, during the first 6 months, on death/ESRD. Eligible patients are allocated to one of four regimens: (1) PLEX and standard dose GC; (2) PLEX and reduced dose GC; (3) no PLEX and standard dose GC; and (4) no PLEX and reduced dose GC (Figure 1).

Allocation will be performed using an online web-based system developed by the data coordinating center (Birmingham Clinical Trials Unit, UK) using minimization. The minimization algorithm is not fully deterministic (that is, the probability of allocation to particular group will never be 1. Minimization strata are based on the following factors: severity of renal disease at presentation (requiring dialysis or creatinine ≥500 μmol/L *vs*. <500 μmol/L), age (<60 *vs*. ≥60 years old), ANCA subtype (PR3-ANCA *vs*. MPO-ANCA), severity of lung hemorrhage (no hemorrhage, lung hemorrhage with an oxygen saturation of ≤85% on room air or ventilated, or lung hemorrhage with an oxygen saturation of >85% on room air), and type of induction therapy (oral CYC *vs*. intravenous (IV) CYC *vs*. rituximab).

### Participants

Eligible patients have a diagnosis of active AAV that is consistent with the Chapel-Hill consensus definitions of GPA, MPA or renal limited vasculitis, a positive ANCA to the proteinase 3 or myeloperoxidase serotypes, and a severe manifestation of AAV. A severe manifestation is defined as either renal involvement with a documented estimated glomerular filtration rate <50 mL/min, in addition to hematuria and proteinuria or a renal biopsy demonstrating focal necrotizing glomerulonephritis. Alternatively, patients with lung hemorrhage defined as radiographic evidence in the absence of an alternative explanation in addition to at least one of the following: evidence from broncho-alveolar lavage, frank hemoptysis, an elevation in the diffusing capacity of carbon monoxide, or an otherwise unexplained drop in hemoglobin of at least 2 g/dL.

Patients are excluded if they have concomitant anti-GBM disease, a type of vasculitis other than AAV, are pregnant, required dialysis for at least 21 days prior to randomization, have a renal transplant, have received PLEX within the prior 3 months, have significant CYC exposure, rituximab, or high dose GC prior to randomization, or in the opinion of the treating physician have an absolute indication or contraindication to PLEX or a particular GC regimen or an absolute contraindication to both CYC and rituximab.

Informed consent is obtained from the participant. At centers where surrogate consent is ethically approved, informed consent for participants who lack capacity may be obtained from a surrogate decision-maker.

### Interventions

#### Plasma exchange

PLEX is adjunctive to standard induction therapy and consists of seven exchanges over 14 days of 60 mL/kg per session using 3% to 5% albumin as a replacement solution. Fresh frozen plasma may be used to replace clotting factors in patients at risk of hemorrhage, for example, after renal biopsy or with pulmonary hemorrhage. Other components of the PLEX prescription are determined by local investigator experience and feasibility, including: PLEX modality (centrifugation or filtration), type of anticoagulation, daily or alternate day frequency and route of vascular access. Double filtration (cascade filtration or double membrane filtration) apheresis or immunoabsorption are not permitted.

#### Glucocorticoids

Investigator consensus determined the standard dose regimen of GC (Table 1). The reduced dose regimen provides approximately 55% of the standard dose regimen over the first 6 months. Both regimens are preceded by IV methylprednisolone of between 1 and 3 grams. Oral GC may be provided as prednisolone or prednisone according to local practice.

**Table 1:** Glucocorticoid dosing in the standard and reduced-dose groups of PEXIVAS

**Week**	**Standard**			**Reduced-dose**		
	<50 kg	50-75 kg	>75 kg	<50 kg	50-75 kg	>75 kg
	Pulse	Pulse	Pulse	Pulse	Pulse	Pulse
1	50	60	75	50	60	75
2	50	60	75	25	30	40
3-4	40	50	60	20	25	30
5-6	30	40	50	15	20	25
7-8	25	30	40	12.5	15	20
9-10	20	25	30	10	12.5	15
11-12	15	20	25	7.5	10	12.5
13-14	12.5	15	20	6	7.5	10
15-16	10	10	15	5	5	7.5
17-18	10	10	15	5	5	7.5
19-20	7.5	7.5	10	5	5	5
21-22	7.5	7.5	7.5	5	5	5
23-52	5	5	5	5	5	5
>52	Investigators’ Local Practice			Investigators’ Local Practice		

#### Other treatments

Patients are treated with either oral or IV CYC, or rituximab, as induction immunosuppression according to local practice, patient needs, and physician preference. CYC doses are reduced for advanced age and reduced renal function. Infection and osteoporosis prophylaxis, and blood pressure management are left to the discretion of the local investigator.

### Outcomes

The primary outcome is the time to the composite of death from any cause and ESRD. ESRD is defined as the requirement for at least 12 consecutive weeks of renal replacement therapy (hemodialysis, peritoneal dialysis, and/or continuous renal replacement therapy) or renal transplantation.

Secondary outcomes include death from any cause and ESRD separately, health-related quality of life as measured by the Physical Component and Mental Component Scores of the Short-Form 36 and EuroQoL EQ-5D index score, serious infections, number of serious adverse events, and proportion with a sustained remission. A sustained remission is defined as the achievement of remission prior to 26 weeks and lasting until at least week 52.

### Blinding

This is an open label trial. Blinding of both patients and investigators to PLEX by using a sham PLEX procedure was not considered feasible for this trial. Due to the complexity of the GC regimen it was not feasible logistically or financially to blind the glucocorticoid intervention. To minimize the potential for treatment contamination, the use of non-randomized PLEX for severe, refractory disease must be discussed with the study medical monitor and investigators are provided active feedback on adherence to the GC protocol during case report form completion. Similarly, co-interventions in AAV are limited as patients are uniformly treated for the first year, and subsequent co-interventions are unlikely to influence the trial outcomes. Further, the trial outcomes are unambiguous and objective so the lack of blinding is unlikely to create biased outcome reporting.

### Assessments

Participants are identified by referral at each study center. Recruited participants are seen frequently during the first 3 months of the trial (baseline, 2 weeks, 4 weeks, 8 weeks, and 12 weeks) to ensure adherence to the allocated treatments and because adverse events and mortality in the first 3 months are higher than subsequent time periods. Participants are then seen every 3 months to the end of the first year and then every 6 months to the end of the trial.

### Sample size

**Figure 2:**
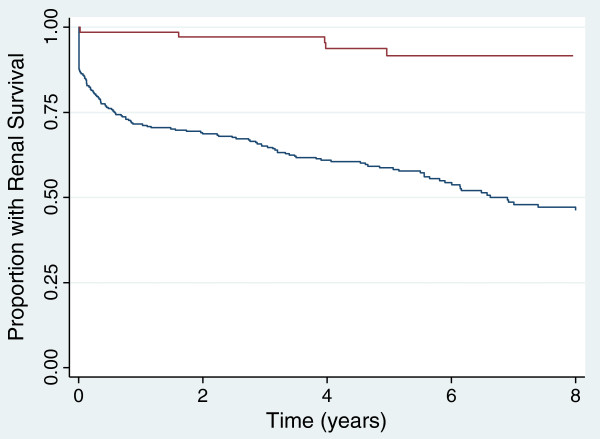
**Differences in long-term renal survival (end-stage renal disease or death) on the basis of estimated glomerular filtration (eGFR) for patients with ANCA-associated vasculitis enrolled in three randomized trials.** Blue line represents patients with an eGFR of ≤ 50 mL/min/1.73 m^2^; red line represents patients with an eGFR of >50 mL/min/1.73 m^2^.

The sample size estimate for this trial assumes a 6-year median time to ESRD or death on the basis of previous extended follow-up studies in randomized trials of AAV of a similar severity to those targeted in this study (Figure 2). Enrollment is estimated to take 5 years with a common close out after the last patient has been followed for 2 years, hence a maximum follow-up of 7 years. Using time-to-event analysis and allowing a 10% loss to follow-up in both groups, 490 patients are required to detect a hazard ratio of 0.64 with 80% power and a two-sided alpha of 0.05. We intend to enroll 500 patients. This predicts approximately 164 events over the study period and is equivalent to a 12% absolute risk reduction of the primary end-point at 5 years (44% in the control group *vs.* 32% in PLEX group). These calculations assume no significant interaction between the two treatment factors. Although this absolute risk appears larger than is often clinically significant, the expensive and invasive nature of PLEX warrants a relatively large effect size.

While this effect size appears reasonable to detect a moderately large effect for PLEX, it is unlikely a reduction in GC will result in a 12% absolute risk reduction of death or dialysis. However, we expect approximately 25% of patients to experience a severe infection based on prior studies. A sample size of 500 patients will allow 80% power to detect at least a 10% absolute risk reduction in severe infections (relative risk reduction of severe infection by 40%), a finding of clinical significance. In terms of the non-inferiority hypothesis, a sample size of 500 patients would allow 80% power to ensure that the reduced dose GC regimen results an increase in ESRD or death by no more than 11% (one-sided alpha of 0.05). However, given dose changes are permitted in response to the clinical events that likely mediate the effects of GC on death and ESRD (that is, uncontrolled disease and infections), it is unlikely that one regimen is substantially inferior to the other in terms of the composite end-point.

### Data management

Data collection is facilitated by either paper-based case report forms or secure electronic case report forms completed online. Research coordinators and/or investigators at each site complete the case report forms, and check them for accuracy and completeness by reviewing source documents and through regular interaction with the participants clinical care providers. Any queries arising from missing data or data anomalies will be resolved.

#### Data monitoring committee (DMC)/data safety and monitoring board (DSMB)

The DMC/DSMB is composed of three members: an expert in vasculitis, a statistician, and an expert in clinical trials (chair). Either trial intervention may be discontinued in the event of clear evidence of harm or benefit. The Haybittle-Peto approach of 3 standard errors will be used as a guideline for a recommendation to stop early at an interim analysis.

### Analyses

All primary analyses will evaluate all patients according to their allocated treatment group. Since a true, qualitative interaction is unlikely we will assume there is no interaction between PLEX and GC regimen unless there is substantial evidence and rationale for the contrary at the time the primary analyses are undertaken. As such, all primary analyses will be at the margins (that is, PLEX *vs*. no PLEX will be evaluated independently from, but stratified by, standard *vs*. reduced dose glucocorticoid regimen and *vice versa*). Point estimates and their corresponding 95% confidence intervals and *p* values will be calculated for all estimates of effect. A *p* value of <0.05 will be considered statistically significant without adjustment for multiple testing.

The time to the composite end-point of death and ESRD as well as death and ESRD individually will be analyzed by time-to-event methods. We will also assess the proportion of the patients that experience the composite outcome by the end of the first year.

We will also perform analyses according to the following subgroups: severity of renal disease at presentation (requiring dialysis or creatinine ≥500 μmol/L *vs*. <500 μmol/L); age (<60 *vs*. ≥60 years old); ANCA binding specificity (PR3 *vs*. MPO); severity of lung hemorrhage (no hemorrhage, hemorrhage with blood oxygen saturation >85% on room air, or hemorrhage with blood oxygen saturation ≤85% on room air); induction immunosuppression therapy used (IV CYC *vs*. oral CYC *vs*. rituximab. Additionally, due to the potential that the investigational treatments may largely affect early mortality and renal function, analyses of the primary outcome (the composite of death and ESRD) will also be performed after censoring data at 12-month follow-up.

## Discussion

PEXIVAS will provide important insights on two treatment strategies for patients with severe AAV. PEXIVAS is also noteworthy in several respects. Considering AAV is a rare disease, PEXIVAS is a relatively large trial and is the largest trial conducted or planned in AAV, or any form of vasculitis, to date. As such, it plans to recruit from between 60 and 90 centers worldwide and has capitalized on existing networks including the European Vasculitis Study Group, the Vasculitis Clinics Research Consortium, and the Australasian Kidney Trials Network. Enhanced network development will have benefits beyond PEXIVAS, including biomarker development and the conduct of further randomized trials in vasculitis. Given the scope of this undertaking, a two-by-two factorial design enhances the efficiency of evaluating important therapies for this disease.

PEXIVAS will investigate two old, well-known therapies for which there is substantial practice pattern variation. Further, one of these therapies is expensive and invasive in that it requires an extracorporeal circuit and represents an additional therapy to improve the efficacy of early treatment (PLEX) while the other is inexpensive, and is a de-escalation of therapy to reduce toxic side effects of early treatment (reduced dose regimen of glucocorticoids). PEXIVAS is also the first trial to actively seek and randomize patients with lung hemorrhage, a manifestation of AAV that is understudied and for which there is no high quality evidence to guide treatment.

## Trial status

PEXIVAS first received ethical approval from the Outer West London Research Ethics Committee of the UK National Research Ethics Service on November 5, 2009 (reference number 09/H0709/56). As of February 22, 2013 PEXIVAS is actively recruiting in 67 centers with additional centers planned. A total of 219 of the planned 500 participants are recruited (http://www.birmingham.ac.uk/research/activity/mds/trials/bctu/trials/renal/pexivas/investigators/recruitment.aspx).

## Abbreviations

AAV: Anti-neutrophil cytoplasm antibody associated vasculitis; ANCA: Anti-neutrophil cytoplasm antibody associated vasculitis; CYC: Cyclophosphamide; DMC: Data monitoring committee; DSMB: Data safety monitoring board; ESRD: End-stage renal disease; GBM: Glomerular basement membrane; GC: Glucocorticoids; GPA: Granulomatosis with polyangiitis; IV: Intravenous; MPA: Microscopic polyangiitis; MPO: Myeloperoxidase; PLEX: Plasma exchange; PR3: Proteinase 3.

## Competing interests

All authors declare no competing interests.

## Authors’ contributions

MW, PM, and DJ contributed to the conception and design of the study, drafting and critical revision of the manuscript, and gave final approval of the version to be published. CAP, CDP and WS contributed to the conception and design of the study, critical revision of the manuscript, and gave final approval of the version to be published. NI, WC, JDZ, LG, KQ, KW, and JLW, contributed to the design of the study, critical revision of the manuscript, and gave final approval of the version to be published. All authors read and approved the final manuscript.

## References

[B1] Watts RA Mooney J Skinner J Scott DG Macgregor AJ The contrasting epidemiology of granulomatosis with polyangiitis (Wegener’s) and microscopic polyangiitis Rheumatology (Oxford) 2012 51 926 931 10.1093/rheumatology/ker454 22258386 PMC3465699

[B2] Flossmann O Berden A De GK Hagen C Harper L Heijl C Hoglund P Jayne D Lugmani R Mahr A Mukhtyar C Pusey C Rasmussen N Stegeman C Walsh M Westman K European Vasculitis Study Group Long-term patient survival in ANCA-associated vasculitis Ann Rheum Dis 2011 70 488 494 10.1136/ard.2010.137778 21109517

[B3] Hoffman GS Kerr GS Leavitt RY Hallahan CW Lebovics RS Travis WD Rottem M Fauci AS Wegener granulomatosis: an analysis of 158 patients Ann Intern Med 1992 116 488 498 1739240 10.7326/0003-4819-116-6-488

[B4] Pagnoux C Hogan SL Chin H Jennette JC Falk RJ Guillevin L Nachman PH Predictors of treatment resistance and relapse in antineutrophil cytoplasmic antibody-associated small-vessel vasculitis: comparison of two independent cohorts Arthritis Rheum 2008 58 2908 2918 10.1002/art.23800 18759282 PMC2754705

[B5] Walsh M Flossmann O Berden A Westman K Hoglund P Stegeman C Jayne D European Vasculitis Study Group Risk factors for relapse of antineutrophil cytoplasmic antibody-associated vasculitis Arthritis Rheum 2012 64 542 548 10.1002/art.33361 21953279

[B6] Seo P Min YI Holbrook JT Hoffman GS Merkel PA Spiera R Davis JC Ytterberg SR St Clair EW McCune WJ Specks U Allen NB Lugmani RA Stone JH WGET Research Group Damage caused by Wegener’s granulomatosis and its treatment: prospective data from the Wegener’s Granulomatosis Etanercept Trial (WGET) Arthritis Rheum 2005 52 2168 2178 10.1002/art.21117 15986348

[B7] Little MA Nightingale P Verburgh CA Hauser T De GK Savage C Jayne D Harper L European Vasculitis Study (EUVAS) Group Early mortality in systemic vasculitis: relative contribution of adverse events and active vasculitis Ann Rheum Dis 2010 69 1036 1043 10.1136/ard.2009.109389 19574233

[B8] Jayne DR Gaskin G Rasmussen N Abramowicz D Ferrario F Guillevin L Mirapeix E Savage CO Sinico RA Stegeman CA Westman KA van der Woude FJ van Wijngaarden RA DL Pusey CD European Vasculitis Study Group Randomized trial of plasma exchange or high-dosage methylprednisolone as adjunctive therapy for severe renal vasculitis J Am Soc Nephrol 2007 18 2180 2188 10.1681/ASN.2007010090 17582159

[B9] Jones RB Tervaert JW Hauser T Luqmani R Morgan MD Peh CA Savage CO Segelmark M Tesar V van Paassen P Walsh D Walsh M Westman K Jayne DR European Vasculitis Study Group Rituximab versus cyclophosphamide in ANCA-associated renal vasculitis N Engl J Med 2010 363 211 220 10.1056/NEJMoa0909169 20647198

[B10] Lockwood CM Rees AJ Pearson TA Evans DJ Peters DK Wilson CB Immunosuppression and plasma-exchange in the treatment of Goodpasture’s syndrome Lancet 1976 1 711 715 56532 10.1016/s0140-6736(76)93089-0

[B11] Walsh M Tonelli M Jayne D Manns B Surrogate end points in clinical trials: the case of anti-neutrophil cytoplasm antibody-associated vasculitis J Nephrol 2007 20 119 129 17514615

[B12] Walsh M Catapano F Szpirt W Thorlund K Bruchfeld A Guillevin L Haubitz M Merkel PA Peh CA Pusey C Jayne D Plasma exchange for renal vasculitis and idiopathic rapidly progressive glomerulonephritis: a meta-analysis Am J Kidney Dis 2011 57 566 574 10.1053/j.ajkd.2010.10.049 21194817 PMC3062650

[B13] Szpirt WM Heaf JG Petersen J Plasma exchange for induction and cyclosporine A for maintenance of remission in Wegener’s granulomatosis–a clinical randomized controlled trial Nephrol Dial Transplant 2011 26 206 213 10.1093/ndt/gfq360 20577017

[B14] van Wijngaarden RADL Hauer HA Wolterbeek R Jayne DR Gaskin G Rasmussen N Noel LH Ferrario F Waldherr R Hagen EC Bruijn JA Bajema IM Clinical and histologic determinants of renal outcome in ANCA-associated vasculitis: A prospective analysis of 100 patients with severe renal involvement J Am Soc Nephrol 2006 17 2264 2274 10.1681/ASN.2005080870 16825335

[B15] van Wijngaarden RADL Hauer HA Wolterbeek R Jayne DR Gaskin G Rasmussen N Noel LH Ferrario F Waldherr R Bruijn JA Bajema IM Hagen EC Pusey CD EUVAS Chances of renal recovery for dialysis-dependent ANCA-associated glomerulonephritis J Am Soc Nephrol 2007 18 2189 2197 10.1681/ASN.2007010066 17596637

[B16] Klemmer PJ Chalermskulrat W Reif MS Hogan SL Henke DC Falk RJ Plasmapheresis therapy for diffuse alveolar hemorrhage in patients with small-vessel vasculitis Am J Kidney Dis 2003 42 1149 1153 10.1053/j.ajkd.2003.08.015 14655185

[B17] Lockwood CM Boulton-Jones JM Lowenthal RM Simpson IJ Peters DK Recovery from Goodpasture’s syndrome after immunosuppressive treatment and plasmapheresis Br Med J 1975 2 252 254 10.1136/bmj.2.5965.252 1131574 PMC1673280

[B18] Pusey CD Rees AJ Evans DJ Peters DK Lockwood CM Plasma exchange in focal necrotizing glomerulonephritis without anti-GBM antibodies Kidney Int 1991 40 757 763 10.1038/ki.1991.272 1745027

[B19] Rota S Rambaldi A Gaspari F Noris M Daina E Benigni A Perna A Donadelli R Remuzzi G Garattini S Methylprednisolone dosage effects on peripheral lymphocyte subpopulations and eicosanoid synthesis Kidney Int 1992 42 981 990 10.1038/ki.1992.377 1453591

[B20] Irakam A Miskolci V Vancurova I Davidson D Dose-related inhibition of proinflammatory cytokine release from neutrophils of the newborn by dexamethasone, betamethasone, and hydrocortisone Biol Neonate 2002 82 89 95 10.1159/000063094 12169830

[B21] Stone JH Merkel PA Spiera R Seo P Langford CA Hoffman GS Kallenberg CG St Clair EW Turkiewicz A Tchao NK Webber L Ding L Sejismundo LP Mieras K Weitzenkamp D Ikle D Seyfert-Margolis V Mueller M Brunetta P Allen B Fervenza FC Geetha D Keogh KA Kissin EY Monach PA Peikert T Stegeman C Ytterberg SR Specks U RAVE-ITN Research Group Rituximab versus cyclophosphamide for ANCA-associated vasculitis N Engl J Med 2010 363 221 232 10.1056/NEJMoa0909905 20647199 PMC3137658

[B22] Saag KG Koehnke R Caldwell JR Brasington R Burmeister LF Zimmerman B Kohler JA Furst DE Low dose long-term corticosteroid therapy in rheumatoid arthritis: an analysis of serious adverse events Am J Med 1994 96 115 123 10.1016/0002-9343(94)90131-7 8109596

[B23] Wolfe F Caplan L Michaud K Treatment for rheumatoid arthritis and the risk of hospitalization for pneumonia: associations with prednisone, disease-modifying antirheumatic drugs, and anti-tumor necrosis factor therapy Arthritis Rheum 2006 54 628 634 10.1002/art.21568 16447241

[B24] Illei GG Yarboro CH Kuroiwa T Schlimgen R Austin HA Tisdale JF Chitkara P Fleisher T Klippel JH Balow JE Boumpas DT Long-term effects of combination treatment with fludarabine and low-dose pulse cyclophosphamide in patients with lupus nephritis Rheumatology (Oxford) 2007 46 952 956 10.1093/rheumatology/kem001 17317716

[B25] Knight SR Morris PJ Steroid avoidance or withdrawal after renal transplantation increases the risk of acute rejection but decreases cardiovascular risk. A meta-analysis Transplantation 2010 89 1 14 10.1097/TP.0b013e3181c518cc 20061913

[B26] Pascual J Galeano C Royuela A Zamora J A systematic review on steroid withdrawal between 3 and 6 months after kidney transplantation Transplantation 2010 90 343 349 20574419 10.1097/TP.0b013e3181e58912

[B27] Griggs RC Batshaw M Dunkle M Gopal-Srivastava R Kaye E Krischer J Nguyen T Paulus K Merkel PA Rare Diseases Clinical Research Network Clinical research for rare disease: opportunities, challenges, and solutions Mol Genet Metab 2009 96 20 26 10.1016/j.ymgme.2008.10.003 19013090 PMC3134795

